# A Wearable In‐Pad Diagnostic for the Detection of Disease Biomarkers in Menstruation Blood

**DOI:** 10.1002/advs.202505170

**Published:** 2025-05-24

**Authors:** Lucas Dosnon, Thomas Rduch, Charlotte Meyer, Inge K. Herrmann

**Affiliations:** ^1^ Nanoparticle Systems Engineering Laboratory Institute of Energy and Process Engineering (IEPE) Department of Mechanical and Process Engineering (D‐MAVT) ETH Zurich Sonneggstrasse 3 Zurich 8092 Switzerland; ^2^ Particles Biology Interactions Laboratory Department of Materials Meet Life Swiss Federal Laboratories for Materials Science and Technology (Empa) Lerchenfeldstrasse 5 St. Gallen 9014 Switzerland; ^3^ Department of Gynecology and Obstetrics (Frauenklinik) Cantonal Hospital St. Gallen (KSSG) Rorschacherstrasse 95 St. Gallen 9007 Switzerland; ^4^ The Ingenuity Lab University Hospital Balgrist Forchstrasse 340 Zurich 8008 Switzerland; ^5^ Faculty of Medicine University of Zurich Rämistrasse 100 Zürich 8006 Switzerland

**Keywords:** biomarkers, blood, menstruation, non‐invasive, wearable, women's health

## Abstract

The pain‐free monitoring of blood‐based biomarkers is essential for early detection of diseases like cancers, infections, and metabolic disorders such as diabetes. While research often focuses on venous blood analysis, menstruation blood is an overlooked but promising source. Evidence shows a strong correlation between biomarker levels in menstruation and venous blood for many clinical analytes. A wearable, microfluidic monitoring platform integrated into hygiene pads is presented for electronic‐free, naked‐eye detection of disease biomarkers in menstruation blood (MenstruAI). Semi‐quantitative detection of C‐reactive protein (CRP), cancer biomarkers carcinoembryonic antigen (CEA) and cancer antigen 125 (CA‐125), and endometriosis biomarker CA‐125 is demonstrated. The biomarker‐induced color changes can be read by the naked eye or a smartphone app with a machine‐learning algorithm for semi‐quantitative analysis. MenstruAI can revolutionize women's health by offering a non‐invasive, affordable, and accessible health monitoring method, democratizing healthcare, and enhancing service availability and equity.

## Graphical abstract

MenstruAI integrates a paper‐based biosensor into sanitary pads to detect biomarkers in menstrual blood, enabling accessible, lab‐free diagnostics health. The platform supports early disease detection, especially in underserved communities, while challenging menstrual stigma and opening pathways for scalable, inclusive, and sustainable population health screening through wearable technologies.

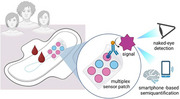

## 1 Introduction

In modern medicine, blood tests and blood panels are indispensable, providing valuable insights into various physiological aspects to make informed decisions about patient care. The levels of molecular constituents in blood are directly associated with the physiological state of the body.^[^ ^1^, ^2^, ^3^, ^4^ ^]^ Traditionally, blood testing has been confined to laboratory environments, necessitated by the requirement for sample pre‐processing, intricate analytical methods, and specialized instrumentation.^[^ ^5^ ^]^ However, the diagnostic significance of blood renders it a prime candidate for point‐of‐care (POC) diagnostic applications.^[^ ^6^ ^]^ While lateral flow assay (LFA)‐based analyses of saliva or urine are well‐established, the analysis of whole blood samples, which contain many more biomarkers, typically requires invasive blood collection as well as intricate sample preprocessing.^[^ ^7^ ^]^ Capillary blood samples obtained through finger pricks are most frequently used.^[^ ^8^ ^]^ Nonetheless, blood collection is invasive, leading to poor compliance and reluctant adoption by both patients and, even more so, seemingly healthy individuals. Additionally, using blood in point‐of‐care devices presents increased barriers to reproducibility and ease of use due to coagulation that hinders its usage, or simply the interference from the red color that may alter a colorimetric readout on a paper‐based sensor.^[^ ^9^ ^]^ As a result, most of the research conducted on blood‐based point‐of‐care devices involves the use of processed blood. This includes blood containing anticoagulants, blood that has been diluted beforehand with a running buffer, or blood that has been separated from certain components, such as red blood cells, to use only the remaining plasma.^[^ ^10^, ^11^, ^12^, ^13^ ^]^ These intricate processing steps pose a significant barrier to regular, low‐cost health monitoring based on blood biomarkers. While a major focus of the research community lies in investigating pain‐free blood sampling and devices for venous blood analysis, menstrual blood appears to be a largely ignored sampling source. Even though every month, 1.8 billion people menstruate,^[^ ^14^ ^]^ menstruation blood is largely under‐represented in both basic and translational research.^[^ ^15^ ^]^ Interestingly, menstrual blood has shown a considerable correlation with systemic blood in terms of protein content through molecular proteomic analysis. Additionally, it contains 385 unique proteins that open the door to further analysis and understanding of women's health.^[^ ^16^, ^17^ ^]^ As a result, menstruation blood is a promising alternative for the noninvasive collection of blood for diagnosis and health monitoring for the menstruating population. Such an approach may be especially promising and economically viable for resource‐constrained settings where patients have no access to regular check‐ups, as well as diseases with a relatively low prevalence in the population, where preventative testing at the doctor's office is not economically feasible.^[^ ^18^ ^]^ In addition to providing a non‐invasive means of accessing blood, menstrual blood is characterized by lower concentrations of coagulation factors, along with reduced levels of hemoglobin and hematocrit.^[^ ^19^ ^]^ These properties render menstrual blood particularly well‐suited for point‐of‐care testing, circumventing the need for the preprocessing steps often necessary in the analysis of venous blood.

Recent studies have explored the utilization of menstrual blood for health monitoring purposes, such as for glycated hemoglobin (HbA1c) measurement in diabetic patients^[^ ^20^ ^]^ or for human papillomavirus (HPV) detection.^[^ ^21^ ^]^ Such studies leveraged the use of menstrual blood for diagnostics purposes as compared with venous blood and highlighted the strong concordance of biomarkers levels between the two samples. They also support the utility of a device that would use menstruation blood to provide medical diagnostics. However, these measurements were performed from menstrual pad dried blood spots that were eluted and processed in centralized analytical laboratories. Access to such facilities is not universally available, making the process complex, burdensome and inaccessible for many. While some concepts of sanitary pad‐based sensors were presented, they only focused on aspects such as volume control of blood loss^[^ ^22^ ^]^ and pH levels^[^ ^23^ ^]^ and did not leverage menstrual blood for gaining valuable insights into the physiological state of an individual. Conceptualizing biomarker detection and monitoring methods that do not rely on expensive analytical processes would therefore address unmet needs, providing a more accessible and practical solution.

**Figure 1:**
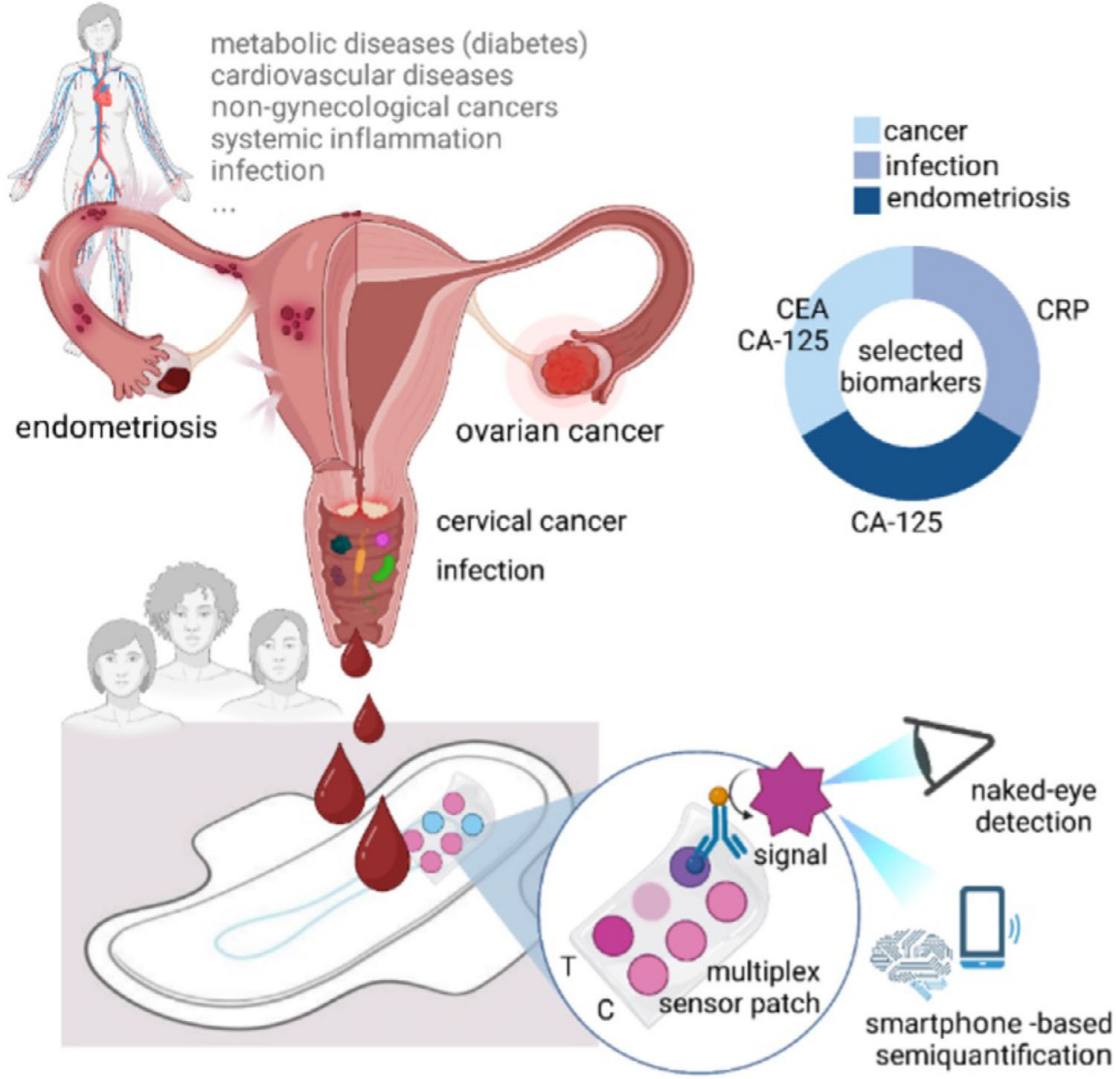
Concept of menstruation blood biosensor integrated into hygiene pads enabling direct naked‐eye readable semi‐quantitative analysis of biomarkers in unprocessed menstruation blood. The platform termed MenstruAI is designed to include infection/inflammation, cancer and endometriosis biomarkers based on an in‐pad integrated lateral flow assay (LFA) platform with integrated volume control.

In this work, we report the design and development of a non‐invasive low‐cost paper based diagnostic platform for the effortless direct on‐pad detection of disease biomarkers in menstruation blood by naked‐eye detection or smartphone‐app (termed MenstruAI), not requiring pre‐ or post‐processing (**Figure** **1**). We demonstrate direct semi‐quantitative detection of a panel of biomarkers that are often associated with infection and inflammation (C‐reactive protein), gynecological cancers (CEA and CA‐125) and gynecological disorders such as endometriosis (CA‐125) in menstrual blood. Therefore, this first‐of‐its‐kind point‐of‐care device enables completely passive (not involving human intervention other than deciding to use MenstruAI pads) and reproducible biomarker measurements directly in unprocessed menstruation blood. To assist users in interpreting their results, we introduce a smartphone app that guides them through using the sensor to obtain and interpret test results. This app also enables sharing the results with healthcare professionals with permission of the user, enabling them to make informed decisions in the event of any significant abnormalities. The MenstruAI platform directly promotes the use of a sample often seen as waste and increases the accessibility of women to cutting‐edge technologies centered on women's health. It may signal to the user that additional checks at a doctor's office may be advisable, and with it have transformative impact on women's health by offering a non‐invasive and barrier‐free route to close‐meshed health monitoring.

## 2 Results and Discussion

### 2.1 Establishment of LFA for CEA, CA‐125, and CRP in Serum and Whole Blood

To address the unmet need for affordable and accessible women's health diagnostics, we sought to design a wearable for the reliable, multiplex detection and semi‐quantification of health‐relevant analytes in menstrual blood. The solution is designed to provide an accessible, affordable, reproducible, non‐invasive method for detecting important health biomarkers through menstrual blood, offering a convenient, comfortable, and accessible solution for personal health monitoring to the menstruating population.

First, LFA sensors were designed for the detection and semi‐quantification of individual biomarkers in relevant biofluids (including whole blood) and clinically relevant detection windows. CEA, CA‐125 and CRP were selected as representative biomarkers, covering diagnostically interesting areas of cancer, endometriosis, and infection, making them particularly relevant for women's health. Additionally, the presence of these biomarkers is indicative of a pathology since baseline levels in healthy individuals (even during menstruation^[^ ^24^, ^25^ ^]^) are typically low and in the region of the analytical detection limits of a typical LFA. Careful optimization of the LFA sensors was performed to achieve detection and semi‐quantification in i) human serum, and ii) unprocessed human whole blood for practicality, ease of use and to eliminate the need for sample transformation or addition of a running buffer. The average coefficients of variation (CV) across all replicates of LFA for each biomarker were <10%. The LFAs were engineered to incorporate a test line, a control line, and, in the case of CRP detection, an antigen line. This design uses a competitive assay format to circumvent the so‐called hook effect,^[^ ^26^ ^]^ which otherwise hinders the detection of CRP at levels above 10 µg mL^−1^,^[^ ^27^, ^28^, ^29^, ^30^, ^31^ ^]^ thereby limiting its detection within a clinically relevant range.

Antibody‐functionalized gold nanoparticles (AuNP) were used as recognition element for the different analytes. During the development and analysis of LFA, a sufficient volume of a spiked fluid sample (i.e. serum or whole blood obtained from volunteers was applied to the sample pad of the LFA. This fluid sample subsequently migrated towards the conjugate pad on the LFA, where the target proteins within the sample interacted with functionalized gold nanoparticles pre‐deposited on the conjugate pad via antigen‐antibody reaction. The test line on the nitrocellulose (NC) membranes consisted of antibodies designed to capture these protein‐nanoparticle complexes, thus facilitating a sandwich reaction that resulted in the visualization of the test line. A Fusion 5 blood separation membrane was introduced to effectively filter out red blood cells, successfully retaining them within the glass fibers of the sample separation pad. This selective filtration ensured that only plasma progressed through the assay. Fusion 5 separates up to 50 µL of blood per cm^2^ with a plasma recovery rate of 90 to 95%. Consequently, the red hue typically associated with blood was removed, allowing for a clear and unaltered measurement of color intensity at the test and control lines.

### 2.2 Direct Measurement of CEA Level in Serum and Whole Blood

**Figure 2:**
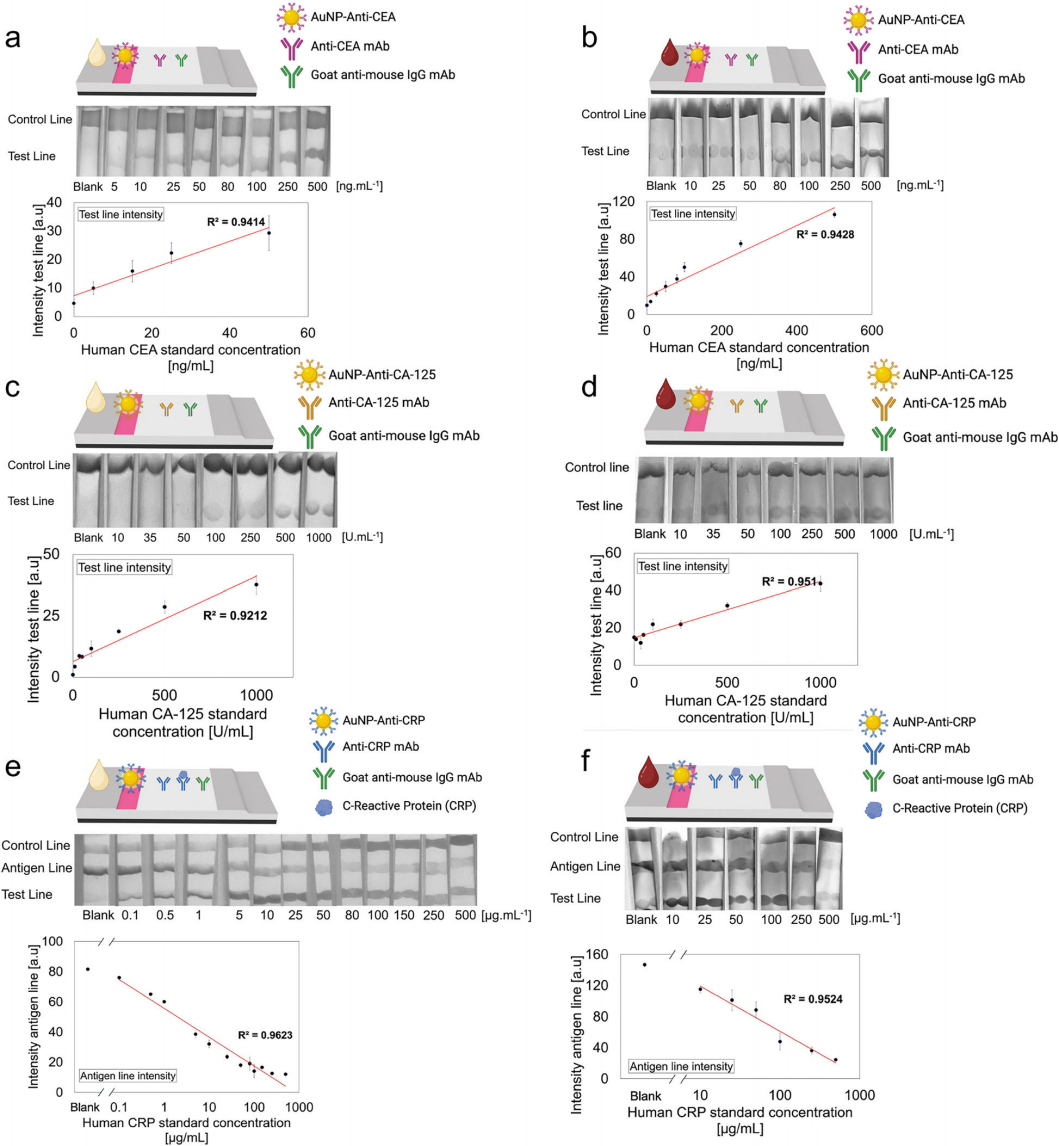
Design of LFAs suitable for biomarker detection in human serum and whole blood in clinically relevant detection windows. a) Design of CEA LFA assay using human CEA‐antibody functionalized 40 nm gold nanoparticles in human serum and b) in fresh, unprocessed human whole blood. c) Design of CA‐125 LFA assay using human CA‐125‐antibody functionalized 40 nm gold nanoparticles in human serum and d) in fresh, unprocessed human whole blood. e) Design of CRP LFA assay using human CRP‐antibody functionalized 20 nm gold nanoparticles in human serum and f) in fresh, unprocessed human whole blood. Assays were performed in triplicate using independent blood donors (*N* = 3). LFA images shown in grayscale.

CEA was selected as first candidate biomarker relevant in many malignant diseases. Often naturally present at concentrations below 5.0 ng mL^−1^ in healthy individuals, it can be used to indicate the presence of tumors or spreading cancer when reaching values above 20 ng mL^−1^.^[^ ^32^, ^33^ ^]^ Thus, a gold‐based LFA for CEA in the clinically relevant detection window (0‐500 ng mL^−1^) was established using human‐CEA antibodies immobilized on 40 nm spherical gold nanoparticles. The use of 40 nm gold nanoparticles offered the highest and most sensitive signal compared to other sizes of gold nanoparticles tested. A logarithmic response curve was observed with increasing concentration of CEA between 0 and 500 ng mL^−1^ (Figure S1, Supporting Information). A linear response was obtained between 0 and 50 ng mL^−1^ with a linear correlation coefficient R^2^ of 0.94 (**Figure** **2**a). For human whole blood, a linear response was obtained on the entire range of concentration, between 0 and 500 ng mL^−1^ with a coefficient R^2^ of 0.94 (Figure 2b). The limit of detection (LOD) of the assay is 0.85 ng mL^−1^. Taken together, this assay configuration demonstrated a linear response in the diagnostically important concentration range and sufficient robustness for reliable detection of CEA in a diversity of samples, including serum, plasma, and unprocessed human whole blood.

### 2.3 Direct Measurement of CA‐125 Level in Serum and Whole Blood

In addition to CEA, CA‐125 was targeted due to its significance in gynecological disorders. The conventional upper limit of normal CA‐125 in serum is 35 U mL^−1^.^[^ ^34^, ^35^ ^]^ Studies^[^ ^36^ ^]^ showed that 1% of healthy women and 82% of patients with ovarian cancer had CA‐125 levels above this limit. Overexpression of CA‐125 could indicate the presence of potential gynecological cancer or endometriosis.^[^ ^37^, ^38^, ^39^ ^]^ Women suffering from endometriosis have been shown to have an elevated level of CA‐125, reaching values >100 U mL^−1^.^[^ ^40^ ^]^ The clinical range of interest of CA‐125 is relatively large, with values reaching 1000 U mL^−1^ and higher. Therefore, a gold based LFA in the clinically relevant detection window (0–1000 U mL^−1^) was established. Human CA‐125 antibodies were immobilized on 40 nm spherical gold nanoparticles. Similarly, as for CEA, the use of 40 nm gold nanoparticles offered the highest and most sensitive signal compared to other sizes of gold nanoparticles tested. In spiked human serum samples, a linear response was obtained over the range between 0 and 1000 U mL^−1^ with a coefficient R^2^ of 0.92 (Figure 2c). For human whole blood samples spiked with similar concentrations of CA‐125, the linear response was even more satisfying reaching a coefficient R^2^ of 0.95 (Figure 2d). Finally, the LOD of the assay is 2.57 U mL^−1^. This demonstrates the capacity of the LFA to perform CA‐125 detection over the chosen diagnostically relevant range in a both serum and unprocessed human whole blood.

### 2.4 Direct Measurement of CRP Level in Serum and Whole Blood

Finally, CRP was selected as an inflammation biomarker widely used in clinical settings to measure acute inflammation,^[^ ^41^ ^]^ but also more recently to assess the risk of developing cardiovascular disease.^[^ ^42^ ^]^ CRP is very often used as a model analyte for LFA. However, it is rarely measurable over the full CRP concentration range (0 to 500 µg mL^−1^) due to the so‐called “hook effect”.^[^ ^26^ ^]^ The hook effect is a known effect in immunoassays that occurs when high concentrations of analytes lead to decreased signal intensity due to the saturation of binding sites on the test line. The formation of the antigen‐antibody complex is prevented, resulting in a false‐negatives. We designed a three‐line LFA geometry, incorporating a sandwich assay‐based line that allows to perform the detection of CRP between 0 and 10 µg mL^−1^ (Figure S2a–c, Supporting Information). Additionally, a competitive assay‐based line with the pre‐immobilized antigen was added to counter the hook effect that takes place for the detection of CRP level at concentrations above 10 µg mL^−1^ (Figure S2d, Supporting Information). The competitive assay‐based line comprises of the antigen already bound to pre‐immobilized CRP antibody. Thus, in the presence of low concentrations of CRP in the sample, this line has the highest signal intensity, as most AuNP conjugates will get immobilized on it. At high concentrations of CRP, most of the proteins will bind to the AuNP conjugates, which will therefore not bind to the antigen deposited on the line leading to a gradual decrease of the signal intensity (Figure 2e,f). In both human serum and human whole blood samples, introduction of an antigen line led to a very satisfying linear detection range of CRP throughout a clinically relevant window with R^2^ coefficients of 0.96 (0.1–500 µg mL^−1^) and 0.95 (10–500 µg mL^−1^), respectively (Figure 2e,f). This three lines LFA geometry therefore enabled the detection of CRP throughout the full concentration range (0 to 500 µg mL^−1^) with a LOD of 0.15 µg mL^−1^. For all measurements, readout of the sensor was robust and the intensity of the LFA line remained unchanged for at least one year (Figure S3, Supporting Information).

### 2.5 Design of Soft‐Silicon Embedded Paper‐Based Wearable Sensors with Integrated Volume Control

Leveraging the developed LFA sensing technology, we investigated design strategies for its seamless integration into sanitary products. This integration relies on embedding LFA biosensors into hygiene pads, enabling effortless direct, on‐pad analysis of menstrual blood. This approach is guided by several critical design principles, including the simplification of processing steps prior to readout, ensuring minimal procedural complexity. The device's hallmark is its ability to operate autonomously, necessitating no more than a simple selection of the appropriate pad by the user, thanks to the fully integrated LFA technology.

In line with this principle, our wearable prototypes were specifically engineered to analyze unprocessed human menstrual blood for user‐friendly, efficient health monitoring. However, this ambition comes with challenges, notably in ensuring consistent, reproducible sampling amidst the dynamic variability of menstrual blood loss, influenced by individual and physiological factors. In addition to these technical considerations, the integration must meet critical requirements of biocompatibility, comfort, and user‐friendliness. To meet the aforementioned design specifications, we designed two different prototypes, relying on distinctly different volume control approaches.

**Figure 3:**
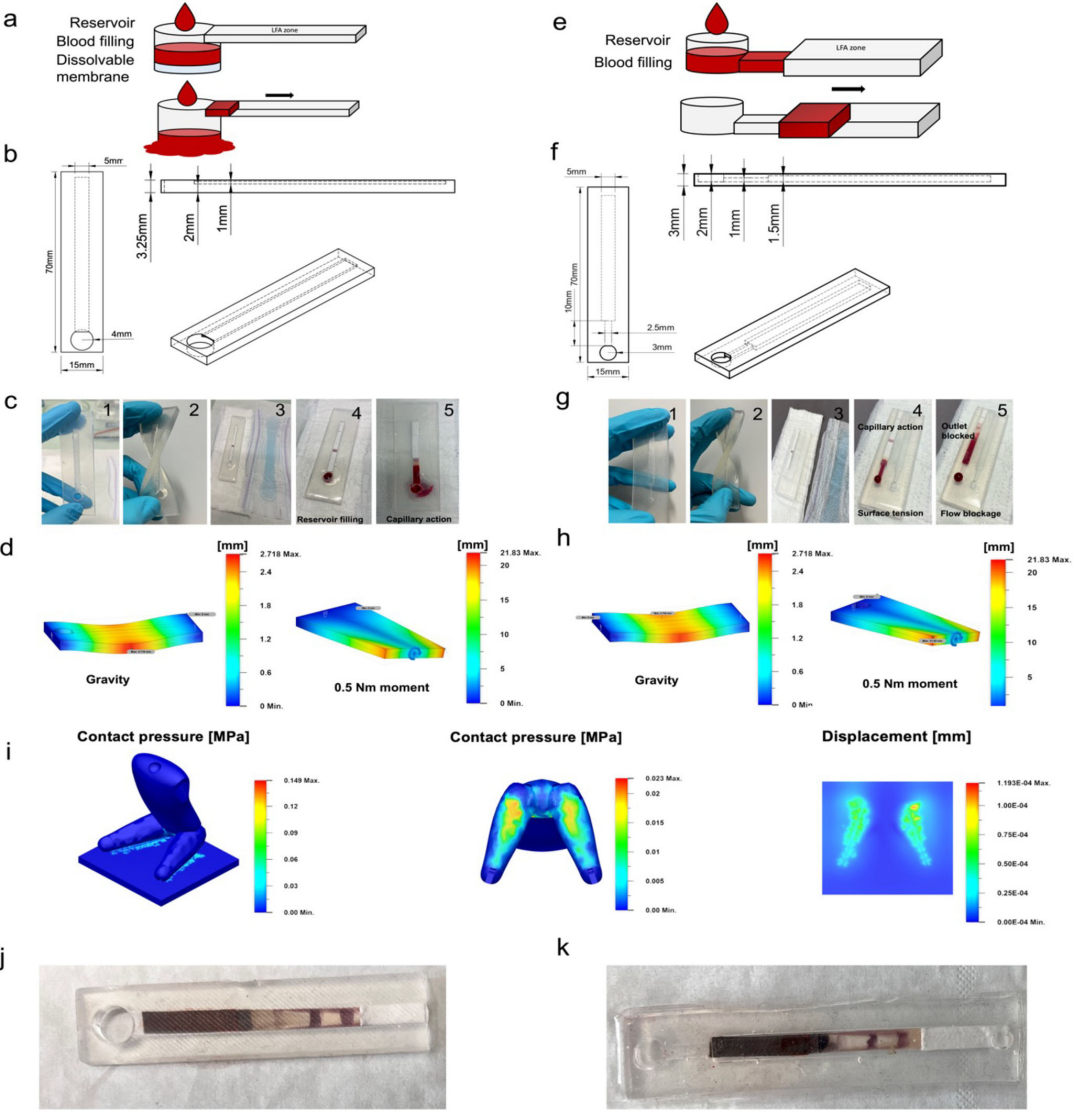
Concept of soft‐silicon casings embedded paper‐based wearable sensors and wearability simulations. a) Concept and b) technical characteristics of prototype design 1 of soft‐silicon embedded wearable paper‐based sensor. The design includes a reservoir of known volume with a dissolvable membrane acting as a drain after test completion. c) Work principle of prototype 1 (1,2: soft‐silicon casing for paper‐based sensor embedding. 3: device placement into sanitary pad. 4,5: capillary‐based intake of controlled volume and LFA test completion. d) Wearability assessed based on mechanical response simulations (displacement) of prototype 1 using gravity and a 0.5 nm moment. e) Concept and f) technical characteristics of prototype design 2 of soft‐silicon embedded paper‐based sensor. The design incorporates an inlet reservoir connected to a capillary channel with predefined dimensions. It utilizes a pressure gradient between the inlet and outlet to precisely control the volume and flow of blood sampled until the correct amount reaches the embedded Lateral Flow Assay (LFA) sensor. g) Work principle of prototype 2. (1,2: soft‐silicon casing for paper‐based sensor embedding. 3: device placement into sanitary pad. 4,5: capillary‐based intake of controlled volume, pressure gradient cancelation, and LFA test completion. h) Wearability assessed based on mechanical response simulations (displacement) of prototype 2 using gravity and a 0.5 nm moment. i) Bodyweight pressure distribution simulation of a female body sitting on a flat surface showing main pressure distribution and displacement at the ischial tuberosities. j) Resulting soft‐silicon casing prototype 1 embedded LFA after it has been removed from a sanitary pad worn by a volunteer. k) Resulting soft‐silicon casing prototype 2 embedded LFA after it has been removed from a sanitary pad worn by a volunteer.

Prototype 1 was designed with a soft‐silicon casing that encases an LFA, featuring an inlet reservoir with a time‐dissolvable polyvinylpyrrolidone (PVP) membrane at its bottom, capable of holding 150 µL of blood for a set duration (**Figure** **3**a). While the PVP membrane is intact, the blood stays in the reservoir, and the LFA test can run to completion. As blood fills the reservoir, it interacts with the PVP membrane, causing it to degrade and eventually fail, a process adjustable through the membrane's PVP concentration (Figure S4, Supporting Information). When the membrane fails, any additional blood simply passes through the now open reservoir and is absorbed by a layer beneath the casing. While the degradation kinetics may vary based on pH, only minor pH changes are to be expected (in absence of a fulminant bacterial infection) thanks to the strong buffering capacity of blood.^[^ ^43^, ^44^, ^45^ ^]^ The device was then integrated into a hygiene pad, positioned under a polypropylene (PP) transfer layer that directed blood into the inlet reservoir. As demonstrated experimentally, the first PP transfer layer filters out blood clots and other cells debris. The soft‐silicon casing ensures that only the necessary volume for LFA initiation is sampled, with excess blood absorbed by the lower pad layers due to the PVP membrane's draining capacity. The hydrophobic soft‐silicon casing prevents undesired fluid exposure ensuring readability of the platform. After the test, the PP layer was removed to reveal the LFA's readout zone through the transparent silicon casing. This design enables reliable volume control and straightforward readout of the integrated LFA test. The inlet reservoir's dimensions are adaptable, aligning with the needs of the LFA and ensuring the correct blood volume is used for the test. The modular design effectively manages sample volume, preventing overflow and protecting the test within a clear casing.

Prototype 2 consists of a similar soft silicon‐based casing containing the LFA strip. The design of the device is different from prototype 1 as it relies on principles of capillary pressure‐driven flow microfluidics that allows passive movements of fluids in microchannels (Figure 3e). When a drop of blood is applied on the first PP transfer layer of the hygiene pad, it automatically gets directed above the inlet reservoir that holds a volume of ≈60 µL. Given the micrometric dimension of the device surface tension forces dominate against gravity. This causes the liquid sample to enter the capillary directing the sample towards the LFA. Inside the microfluidic channel of ≈25 µL volume, the Laplace pressure at the liquid/air meniscus interface is the driving force responsible for the movement of the fluid:^[^ ^46^ ^]^

(1) $$\Delta P=2\gamma \cos\theta \left(\frac{1}{w}+\frac{1}{h}\right)$$

with γ denoting the surface tension, θ the contact angle, *w* the width and *h* the height of the rectangular channel. With the atmospheric pressure *P*_0_ at the outlet, this generates the right pressure gradient to drive the fluid to the LFA. Additionally, considering an incompressible and non‐Newtonian fluid in a laminar flow within a cylindrical capillary, it is possible to use Hagen‐Poiseuille equation:^[^ ^47^ ^]^

(2) $$Q=\frac{\pi r^{4}}{8\;\mu L}\Delta P$$

with *Q* the volumetric flow rate, *r*, and *L* the radius and length of the capillary, *µ* the dynamic viscosity of the liquid, and Δ*P* the pressure difference between inlet and outlet.

The Washburn equation^[^ ^48^ ^]^ then gives the filling rate of a channel with constant capillary pressure:

(3) $$L^{2}=\frac{\gamma D\cos\theta t}{4\mu }$$

with *L* the length of channel that is filled at time *t*, γ the surface tension, θ the contact angle, *D* the diameter of the channel, *µ* the dynamic viscosity. This design ensures consistent capillary filling under fixed channel parameters, finely tuned for controlled sample flow to the LFA. Upon contact, the blood is absorbed by the paper‐based sensor through capillary action, completing the test. The wetting of the paper causes the LFA's sample pad within the silicon device to clog, interrupting the air flow between inlet and outlet, and nullifying the pressure gradient, thereby preventing further liquid entry. This mechanism ensures reproducibility and prevents overflow. In addition, we included draining holes beneath the LFA, channeling excess blood into the absorbent layers of the hygiene pad (Figure 3g). Prototype 2 ensures reproducible sampling of the menstruation blood needed to trigger LFA initiation. The first PP transfer layer filters out undesired elements. The soft‐silicon microfluidic casing directs only the desired blood volume to the paper‐based sensor, while clear readout is maintained thanks the hydrophobic nature of the casing. Like Prototype 1, the device's dimensions and consequently the blood volume are adjustable, offering a flexible solution tailored to the specific design requirements. For read‐out, the top PP layer is easily removable, revealing the LFA's readout zone through the clear casing. In the eventual use scenario, the test can be read while changing the pad, where user manipulation is already involved, and right before discarding it into a sanitary bag.

Evaluation of wearability has been conducted based on several elements. The soft silicon used in both prototypes demonstrates high flexibility, as validated through comprehensive mechanical stress simulations (Figure 3d,h). Even minor stressors, such as gravity or a mere 0.5 N.m moment, induce noticeable deformations. Minor stressors such as gravity or a mere 0.5 N.m moment used to simulate realistic conditions the device would encounter during normal body movements^[^ ^49^, ^50^ ^]^ induce noticeable deformations. Both the soft silicon casings, operating on capillary pressure‐driven flow microfluidics principles, and the wicking mechanism of the paper‐based sensors are resilient against some mechanical constraints such as bending or light compression.^[^ ^51^, ^52^, ^53^, ^54^ ^]^ Furthermore, both designs ensure that our in‐pad integrated paper‐based sensor can be accommodated into a variety of sanitary products available on the market, thanks to their small size and high flexibility. Finally, the device is designed to remain functional while the user sits. Most of the body weight is supported by the ischial tuberosities while sitting,^[^ ^55^, ^56^, ^57^, ^58^ ^]^ which ensures that direct pressure applied to the sanitary pad in the perineal area remains minimal (Figure 3i; Figure S5, Supporting Information. Furthermore, both designs were evaluated anonymously by volunteers during a wearability study. The volunteers were asked to wear the hygiene pad during the second day of their period, for a minimum of 4 h, in the exact same conditions as they would with a commercially available sanitary pad, with no further restriction. Typical daily activities were performed such as walking, commuting and mild physical activities. Both the functionality of the prototypes regarding blood sampling and biomarkers detection, as well as comfort and ease of use were evaluated. In all cases, blood was sampled by the soft‐silicon microfluidics chips, and the LFA was completed (Figure 3j,k). No contamination was found, ensuring readout capacity. Finally, volunteers evaluated the comfort of the prototype to be optimal, with no difference compared to a commercially available sanitary pad.

### 2.6 Smartphone‐Assisted Semi‐Quantitative Analysis of Biomarkers in Menstruation Blood

With the volume‐controlled in‐pad LFA platform designed, the final crucial step for an accessible health monitoring platform lies in the reliable readout of the test results. LFA results are often subject to subjective interpretations, leading to frequent false positives or negatives^[^ ^59^, ^60^ ^]^ especially when using unprocessed whole blood, which can cause interference such as blood leakage, immobilized red blood cells forming misleading lines, or lysed cells creating a red background. The semi‐quantitative output from multiplexed detection of biomarkers like CEA, CA‐125, and CRP, including an antigen line in the case of CRP, poses additional challenges for visual evaluation by untrained users, underscoring the need for standardized readout methods.

**Figure 4:**
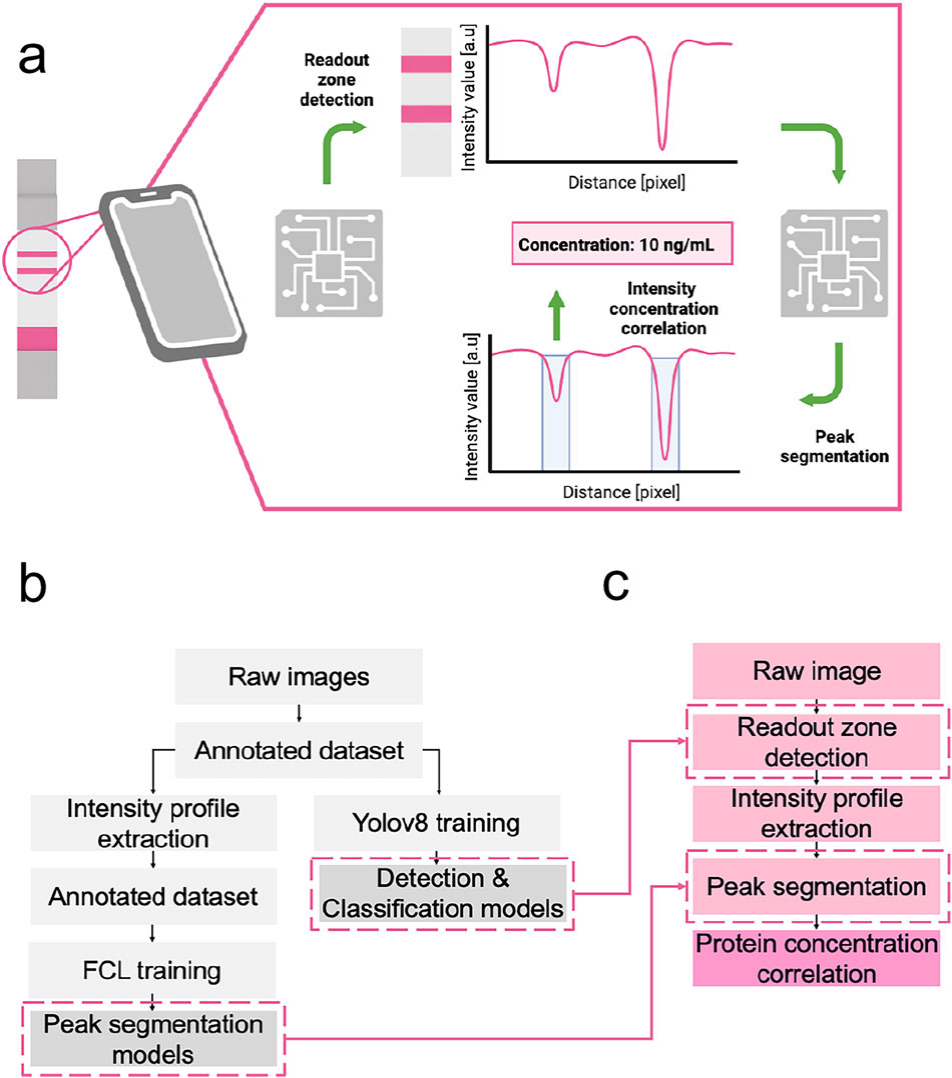
Concept and workflow of MenstruAI machine learning based image analysis software. a) Concept of the smartphone‐based ML for LFA test analysis. A test image was captured with a smartphone. An ML model was employed to identify the readout zone and classify the result, analyzing the pixel intensity profile to locate peaks, corresponding to control and test line intensities. These intensities can be directly linked to biomarker concentration, based on pre‐established integrated calibration. b) Workflow of ML model dataset preparation and training. For the training process, a dataset was first generated using a set of raw images of LFA strips, manually labeled. This dataset was then used for the training of two ML models for readout zone detection and classification. Additionally, intensity profiles were extracted from the same images and manually annotated for peak and background regions. This dataset was then used for the training of the fully connected layer (FCL) model to perform automatic peak segmentation on new (unseen) data. c) Illustration of the workflow of the machine learning application. Once the three models were trained, they were employed to detect and classify the readout zone on new LFA strip images. The intensity profiles were then extracted for peak segmentation and biomarker concentration determination.

**Figure 5:**
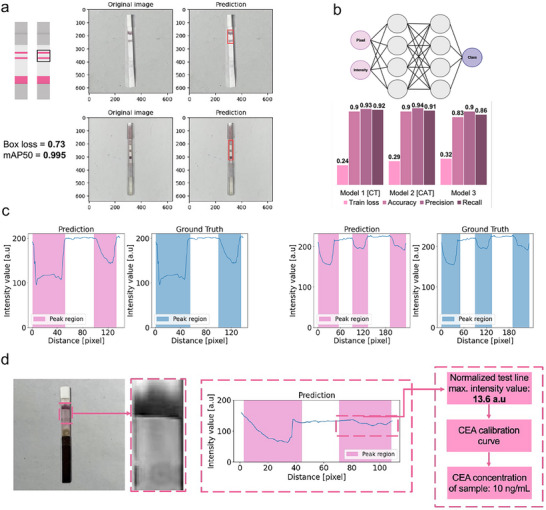
Performances of MenstruAI's machine learning based analysis software. a) Readout zone detection using CNN. The CNN model automatically detected the region of interest where the readout of the test was located. The readout could then be classified. The model reached a box loss of 0.73 and mAP50 of 0.995 after training. b) Concept and performances of FCL models used for peak segmentation. Three models were built similarly with an input layer, hidden layers, and an output layer. Discriminating the output type directly influences positively the performances of the trained models (model 1 and 2) compared to a model trained on all the possible output types (model 3). c) Examples of peak segmentation on intensity profiles displaying 2 and 3 peaks (corresponding to control, test and possibly antigen lines). The FCL model accurately detected the regions corresponding to a peak of intensity on the LFA strips. Comparison between prediction and ground truth demonstrates very satisfactory performances. d) Example use case of automatic CEA quantification from a smartphone‐captured image. The readout zone was accurately detected and classified. Peak segmentation was performed by the FCL model, and the value of CEA was obtained after extraction of the test line intensity, which is linked to the biomarker concentration based on pre‐established calibration.

To address these issues, we developed a fully automatic analysis application using machine learning (ML), deployed on smartphones for standardized assay readouts after pad use. The approach involved using a smartphone‐captured image of the LFA strip, and subsequent analysis with segmentation and classification models (**Figure** **4**). The application first automatically detected the readout zone, where colorful lines (control and test lines for CEA and CA‐125, and control, test, and antigen lines for CRP LFA) were visible. For this purpose, a pre‐trained yolov8 convolutional neural network (CNN) was employed, achieving a box loss of 0.73 and an mAP50 of 0.995 (**Figure** **5**a).

With a confidence threshold of 0.8, it accurately detected the readout zone across the entire test dataset. After segmenting the readout zone, it was processed by a classification CNN and a fully connected layer (FCL) model, enabling peak segmentation on the intensity profile, which displayed 2 or 3 peaks depending on the output type. Without discriminating the output type, the neural network achieved an accuracy, precision, and recall of 0.83, 0.90, and 0.86, respectively. However, when data was classified and separated based on output type, the network's performance increased, with both models’ accuracies reaching 0.90 and precision over 0.93 (Figure 5b). The application then provides the user with the concentration value of the biomarker measured by the LFA, offering a reliable and immediate interpretation of results (Figure 5d).

**Figure 6:**
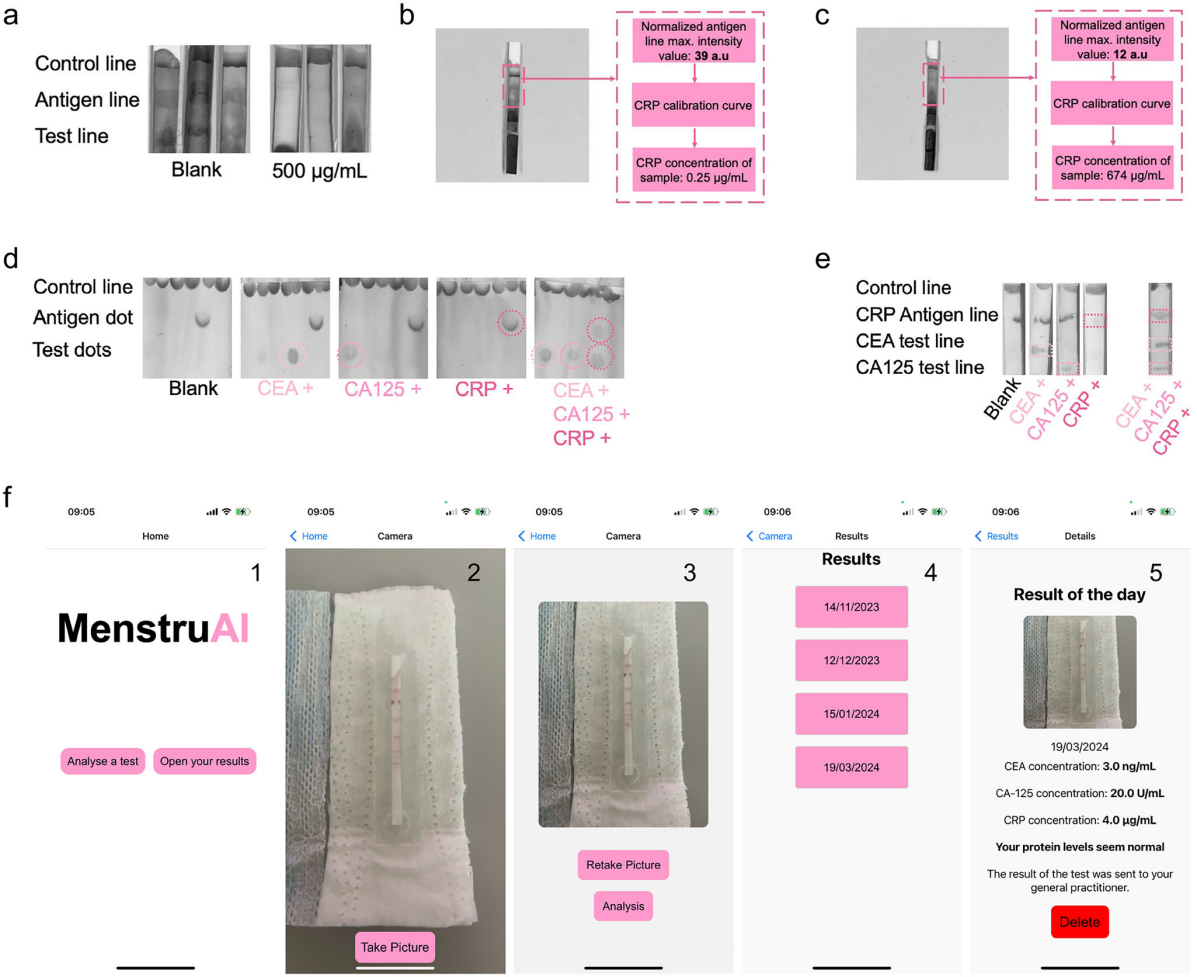
Multiplexed paper‐based sensors for biomarker detection in menstruation blood and MenstruAI's smartphone app analysis. a) LFA analysis of whole menstruation blood containing CRP. Detection and quantification of b) low CRP and c) high CRP in menstruation blood using ML‐assisted readout analysis. d) Multiplexing LFA using a dot‐based design. e) Multiplexing LFA using a line‐based design. f) Representation of the developed smartphone application for test analysis (1: home screen of MenstruAI app. 2: camera screen to acquire an image of the test. 3: validation screen and picture analysis. 4: results screen with precedent test results folders. 5: test result folder with analysis of the image acquired and interpretation of the results). Panels show representative data from tests performed independently at least three times.

Semi‐quantification in menstruation blood was performed using biomarker‐spiked whole menstruation blood (**Figure** **6**a). Reliable biomarker detection and semi‐quantification was obtained using the MenstruAI's smartphone app, with performances similar to detection in venous whole blood (Figure 6b,c). This indicates the feasibility of biomarker detection using the presented LFA platform using unprocessed menstruation blood and MenstruAI's concept.

Additionally, multiplexed paper‐based tests were designed to incorporate the results of the three biomarkers into a single platform. The designs used either a dot‐based (Figure 6d) or line‐based (Figure 6e) readout and enabled concurrent detection of at least three biomarkers (CEA, CA‐125, CRP) with minimal cross‐reaction. Finally, we demonstrate direct on‐pad multiplex biomarker analysis using the software framework (Figure 5) integrated into a smartphone app, allowing ease of use, automatic analysis, and accessibility of the user's data (Figure 6f). The app contains an intuitive and simple user interface, allowing the user to take a picture of the test through the app using the smartphone camera, and analysis of the test either immediately or later. The analysis report with the respective CEA, CA‐125, and CRP menstruation blood levels, along with an interpretation based on the normal clinical range, can then be found alongside precedent reports in the result folder of the app (Figure 6f).

An important aspect of the MenstruAI platform is the assessment of its potential for cost‐effectiveness as a screening tool, assuming the diagnostic performance is validated. The paper‐based sensor technology relies on low‐cost materials, with the cost of a single 6 mm LFA strip approximated at less than $0.40.^[^ ^9^ ^]^ The soft‐silicon casing has been evaluated at $0.50 per unit, bringing the total production cost to approximately $1 per unit. However, for cost‐effectiveness, clinical meaningfulness must be demonstrated by accurately identifying significant biomarker increases and avoiding false positives. While careful optimization of the designs was undergone to ensure reliability of the proposed MenstruAI concept, the conceptual prototypes must be tested in a large field study with many participants to account for the heterogeneity in menstrual blood flow and kinetics which remains largely undocumented. Such a study is essential to ensure the test's useability across a wide panel of users and to identify and address potential limitations inherent to any early‐stage medical device concept. Taken together, MenstruAI concept is compliant with the ASSURED (Affordable, Sensitive, Specific, User‐friendly, Rapid & Robust, Equipment‐free, and Deliverable) criteria determined by the WHO. It is low‐cost and does not require complex instrumentation. It directly promotes the use of a sample readily available and often seen as waste, while ensuring robustness and sensitive detection.

To conclude, in this work, we present a fully integrated on‐pad detection platform for the semi‐quantitative analysis of a panel of model biomarkers relevant for inflammation and infection, cancer, and endometriosis, directly in menstruation blood. This work directly promotes the use of a sample often seen as waste and increases the accessibility of women to cutting‐edge technologies centered on women's health. Additionally, this first‐of‐its‐kind point‐of‐care device features an innovative design that enables fluidic manipulation and multiplexed electronic‐free readout, allowing for completely passive (without active human intervention) and reproducible testing in resource‐constraint settings. This work presents two key innovations, namely the direct analysis of unprocessed, undiluted menstruation blood, and most importantly, fluid‐control mechanisms, that enable accurate dosing of menstruation blood on the sample pad and prevent overflowing and thus invalidation of the test results. The machine‐learning assisted analysis of the LFA assists the user in data interpretation and long‐term tracking of biomarkers. Due to the low‐barrier characteristics of this platform, we expect significant user adoption, and potentially earlier detection of healthy complications. Importantly, the platform in its current form is not intended as a diagnostic replacing clinically established tests, but rather to alert users of potential health issues and enable close‐meshed, affordable health monitoring. In the future, we foresee that the biomarker panel could be extended depending on the user's interest and disease prevalence, potentially also including sexually transmitted disease (STD) biomarkers, etc. The proposed menstruation blood‐based multiplexed detection platform could have a transformative impact on women's health by offering a non‐invasive, affordable and barrier‐free approach to health monitoring in both developed and developing countries, thus contributing to healthcare democratization.

## 3 Experimental Section

### Materials

Sodium citrate dihydrate HOC(COONa)(CH_2_COONa)_2_·2H_2_O, Chloroauric acid HAuCl_4_, Phosphate‐buffered saline (PBS), bovine serum albumin (BSA), sucrose C_12_H_22_O_11_, tween‐20, NaH_2_PO_4_·H_2_O, Na_2_HPO_4_, Na_2_B_4_O_7_·10H_2_O, H_3_BO_3_, polyvinylpyrrolidone (PVP) (C_6_H_9_NO)_n_ were purchased from Sigma‐Aldrich. Anti‐CRP detection and capture monoclonal antibodies were purchased from Abcam inc. (anti‐CRP C2 & anti‐CRP C6). Anti‐CEA detection and capture monoclonal antibodies were purchased from Fitzgerald industries international (anti‐CEA F & anti‐CEA G). Anti‐CA‐125 detection and capture monoclonal antibodies (Anti‐CA‐125 × 325 & Anti‐CA‐125 × 306) were purchased from Lubioscience.

Goat anti‐mouse IgG were purchased from Lubioscience. CRP from human ascites were purchased from Sigma‐Aldrich, and CEA proteins were purchased from Fitzgerald industries international. CA‐125 human protein was purchased from Sigma Aldrich. CRP free human serum was purchased from Lubioscience, and human serum was purchased from Sigma‐Aldrich. Citrate‐phosphate‐Dextrose anticoagulant solution was purchased from Sigma‐Aldrich. All buffers and reagent solutions were prepared using distilled water or MiliQ water (>18.2Ø.cm, Milipore). The sample, conjugate, and absorbent pads, as well as nitrocellulose membranes and adhesive backing cards were obtained as part of a LFA assembly kit purchased from Nanocomposix. Additionally, Fusion 5 blood separation membrane was purchase from Cytiva. Ecoflex near‐clear 00–31 was purchased from Smooth‐On. Sanitary pads were purchased from Always.

### Human Venous and Menstruation Blood Samples

Before testing unprocessed menstruation blood obtained from ten volunteers, two highly abundant, easily accessible biofluids were used to establish the assay working principle for the detection of biomarkers in relevant matrices using the designed LFA. Human serum (Sigma‐Aldrich) was initially used for the assays, while human whole blood was obtained from ten healthy volunteers and used unprocessed. For each assay, spiked samples were tested to establish calibration curves for semi‐quantitative biomarker detection using the MenstruAI platform. After demonstrating feasibility with serum samples, the assay was adapted for use with unprocessed human whole blood obtained from different volunteers and ultimately for the use with fresh human menstruation blood. The collection of venous (BASEC No. 2016‐00816) and menstruation blood (BASEC No. 2023‐00562) was approved by the cantonal ethics commission of the Canton of St. Gallen, Switzerland. Human menstruation blood samples were obtained from ten healthy volunteers on day 1 and 2 of menses. Menstruation blood was collected by the volunteers using a menstrual cup (approved by ethics commission of the canton of St. Gallen, Switzerland) and was donated to a gynecologist at the Cantonal Hospital St. Gallen and handed over to the research lab on the same day in an anonymous way (not transferring donor information).

### Apparatus

A Hidex sense 425‐301 plate reader was used for the absorbance measurement and verification of nanoparticles conjugation. A Prusa extrusion 3D printer was used for 3D printing of parts. An iPhone 13 along with ImageJ software analysis were used for quantitative evaluation of the assay's results.

### Reagents Preparation

Phosphate buffer (0.01M, pH 7.4) was made using Na_2_HPO_4_ and NaH_2_PO_4_.H_2_O in MiliQ water. Borate buffer (100 mm, pH9) was made using Na_2_B_4_O_7_·10H_2_O and H_3_BO_3_ in miliQ water. The sample pad buffer consisted of a solution of PBS containing 0.5% (w/v) BSA and 1% (v/v) tween‐20. Finally, the conjugate pad buffer in which nanoparticles were resuspended after conjugation consisted of a solution of PBS containing 5% (w/v) sucrose, 1% (w/v) BSA, and 0.5% (v/v) tween‐20.

### Gold Nanoparticle Synthesis

To synthesize gold nanoparticles, a 60 mL solution of 2.2 mm of sodium citrate dihydrate was prepared in 3 Neck round bottom glass flask and brought to boiling point. Four hundred microliters of a 25 mm HAuCl_4_ was added afterward, resulting in seed nanoparticles of ≈6 to 10 nm diameter after 15 min. Afterward, a growth step was realized adding 400 µL of a 60 mm sodium citrate dihydrate solution the mix, followed by 400 µL of a 25 mm HAuCl_4_ solution. After cooling down, gold nanoparticles of ≈20 nm diameter were fully synthesized. To obtain larger diameter nanoparticles, additional growth steps were completed until the nanoparticles reached the desired diameter.

### Conjugation of Antibodies to Gold Nanoparticles

To prepare gold nanoparticle conjugates, the desired detection antibodies were passively conjugated to the surface of the nanoparticles from non‐covalent interaction. Briefly, two solutions of 40 nm AuNP at pH9 were respectively mixed with a 300 µg mL^−1^ solution of anti‐CEA antibody diluted in MiliQ water and a 350 µg mL^−1^ solution of anti‐CA‐125 antibody diluted in MiliQ water followed by the addition of 1% BSA in MiliQ water for the obtention of anti‐CEA‐AuNP and anti‐CA‐125‐AuNP conjugates. Similarly, a solution of 20 nm AuNP at pH8 was mixed with a 200 µg mL^−1^ solution of anti‐CRP antibody diluted in MiliQ water followed by the addition of 1% BSA in MiliQ water. After incubation for 30 min, the 40 nm AuNP‐based mixtures and 20 nm AuNP‐based mixtures were respectively centrifuged at 3500 and 10,000 rpm and resuspended in the conjugate pad buffer.

### Lateral Flow Assay Preparation

The LFA sensor for individual biomarker detection consists of four components: a sample pad (blood filtration pad or not), a nitrocellulose membrane, a conjugate pad, and an absorbent pad. The sample pad was pre‐treated with a sample pad buffer to assist in the fluid migration. The desired conjugated nanoparticles in a conjugate pad buffer were deposited on the conjugate pad. The absorbent pad was left untreated. For the CEA‐LFA, a line of a 1 mg mL^−1^ anti‐CEA capture antibody solution in phosphate buffer was deposited on the NC membrane to act as the test‐line, and a 1.0 mg mL^−1^ goat anti‐mouse IgG solution in phosphate buffer was deposited to act as the control line. For the CA‐125‐LFA, a line of a 0.75 mg mL^−1^ anti‐CA‐125 capture antibody solution in phosphate buffer was deposited on the NC membrane to act as the test‐line, and a 1.0 mg mL^−1^ goat anti‐mouse IgG solution in phosphate buffer was deposited to act as the control line. For the CRP LFA, a line of a 0.75 mg mL^−1^ anti‐CRP capture antibody solution in phosphate buffer was deposited on the NC membrane to act as the test‐line. An extra line of a 1.0 mg mL^−1^ anti‐CRP capture antibody solution in phosphate buffer was deposited, followed by the addition of a 1.0 mg mL^−1^ human CRP solution deposited on top of the same line, to act as the antigen line. The control line was formed by a 1.0 mg mL^−1^ goat anti‐mouse IgG in phosphate buffer. All components were dried for 1 h in a 37 °C drying oven, and later assembled on a plastic adhesive backing card. Each segment overlapped its neighboring one by 2 mm. Finally, individual LFA were cut to obtain regular paper‐based sensors of 5 mm width and 6 cm length. The LFA strips were cut manually using a guillotine cutter (Paintersisters Lever cutter A4 compact, purchased from Galaxus.ch)″.

### Lateral Flow Optimization

The selection of antibody concentration, pH of nanoparticle solutions, and nanoparticle concentrations were optimized for each biomarker using a nanoparticle‐bioreceptor aggregation test, a well‐established method to determine the optimal pH and minimum antibody concentration for complete nanoparticle coverage (Figure S6, Supporting Information). Nanoparticle concentration was optimized by varying the optical density (OD) of AuNP solutions applied to the conjugate pad. LFA strips were prepared in triplicate, and the optimal signal intensity and sensitivity determined the final concentration. The selection of 40 nm AuNPs (CEA, CA‐125) and 20 nm AuNPs (CRP) was based on testing different sizes, with the choice made after comparing signal intensity and sensitivity to ensure maximum assay performance. While 20 nm AuNPs provided optimal sensitivity for CRP, 40 nm AuNPs outperformed them for CEA and CA‐125. 40 nm AuNPs offered greater signal intensities, as well as greater signal‐to‐noise ratio (SNR) (Figure S7, Supporting Information). Additionally, the limits of detection for CEA and CA‐125 using 40 nm gold nanoparticles were significantly lower than when using 20 nm gold nanoparticles (0.85 µg mL^−1^ and 2.57 U mL^−1^ against 6.23 µg mL^−1^ and 26.9 U mL^−1^). LODs were extrapolated from the regression using the limit of the blank (μ_*blank*_ + 3*σ_*blank*_). Signal‐to‐noise ratios (SNR) were calculated using the mean intensity and the standard deviation of the noise.

### Test Procedure

Once LFA were prepared, solutions of the biomarker of interest were prepared in different matrices at various concentration. CEA, CA‐125, and CRP solutions were prepared by spiking human serum and CRP‐free human serum, as well as human whole venous blood containing anticoagulant, at desired concentrations. Additionally, CRP solutions were prepared by spiking human whole menstruation blood at desired concentrations. 60 to 100 µL of sample were deposited on the sample pad. The volume corresponds to the adequate amount of fluid necessary to initiate the LFA by capillary action while preventing overflow of the paper‐based test. Once the test was performed, images were obtained using an iPhone 13 and analyzed with ImageJ. The intensity profile of individual color channels of the LFA strip image was obtained, and the peak intensity of the lines was recorded. The intensity value was obtained by subtracting the peak value of the line from the background value. All measurements were triplicated.

### In‐Pad Biosensor Assembly

Fabrication of soft silicon casing started with the design of negative parts and molds in Fusion 360 CAD software, followed by 3D printing with an extrusion 3D printer. Ecoflex near‐clear 00–31 part A and part B were mixed in 1:1 (v/v) ratio. The mix was later poured into the desired mold adding the negative parts to form the desired geometries after curing for 4H at room temperature. The negative parts were later removed manually, and biopsy punchers were used to create inlets and outlets in the silicon casing. The LFA sensors were later integrated into the silicon casing to obtain a fully assembled test platform. PVP membranes for volume control were synthesized by mixing PVP powder in methanol at various concentrations depending on the desired retention time to achieve. Once the mix was fully dissolved, the solution was drop‐cast onto circular‐shaped Teflon mold and left to dry for 1H. Once ready, the PVP membranes were directly added to the silicon casing. A commercial sanitary pad was used as a proof of concept. The three layers composing the pad were separated, and the soft‐silicon device was integrated below the first polypropylene sheet, acting as transfer layer for any fluid encountering the pad. The whole assembly was later used as such for experiments. All simulations were performed using Autodesk fusion 360 (Autodesk). Displacement and Von Mises stress were analyzed. The materials properties used for the bodyweight pressure distribution simulation are described in Figure S5 (Supporting Information).

### Artificial Intelligence Assisted Analysis Using Smartphone App

The smartphone‐based analysis using ArtificiaI Intelligence (AI) had been built using three distinct machine learning models to i) detect the readout zone, ii) classify the type of readout, and iii) perform peak segmentation on the intensity profile of the LFA strip to correlate the test line intensity to the biomarker concentration. The workflow consists of automatically detecting the readout zone on the LFA. The readout zone is the zone where the output of the LFA is visible thanks to AuNP‐based colorful lines. Once the zone is automatically detected, the output was analyzed and classified depending on the type of line visible. A model was used to classify the readout zone in three distinct categories (control line only; test and control lines; test, antigen, and control lines visible). The pixel intensity profile of the region was then obtained and automatically processed with a bespoke model, to extract the value of the test line intensity peak that later correlates with the biomarker concentration using pre‐determined calibration curves.

First, a dataset containing 549 images of individual strips of LFA was built, incorporating LFA for the detection of CEA, CA‐125, and CRP with different fluids (phosphate‐buffered saline, human serum, and blood). The dataset contained images taken under different lighting conditions and angles, and data augmentation was applied.

The dataset had been manually labeled to draw a bounding box containing the AuNP‐based colorful lines on each individual strip. The dataset was divided into training, testing, and validation sets representing 70%, 20%, and 10% respectively. Transfer learning had been leveraged, using a pre‐trained yoloV8 convolutional neural network^[^ ^61^ ^]^ model to learn the detection and segmentation of the readout zone using 50 epochs and a batch size of 10.

In a second time, a dataset for the classification of the readout has been built. 549 images of individual readout zones were cropped out from the images of single LFA strips and separated depending on the readout (control line only; test, and control lines; test, antigen, and control lines visible). The dataset was divided into training, testing and validation sets representing 70%, 15%, and 15% respectively. The same pre‐trained yolov8 CNN model was used to learn the classification of a given LFA readout using 50 epochs and a batch size of 5.

Finally, the automatic peak segmentation was performed using a fully connected layer model. To do so, intensity profiles were manually extracted for each of the 549 individual strips. They were manually labelled, attributing a probability 1 to the pixels displaying a colorful line on the LFA and a probability 0 to the pixels consisting of the background region on the LFA. The labelled profiles were subsequently separated in two: profiles containing test line and control line‐based peaks, and profiles containing test line, antigen line, and control line‐based peaks. For the two types, the dataset was divided into training and testing sets representing 80% and 20% respectively, and two identical FCL models were trained using the pixel position and the corresponding intensity as input for supervised learning of the probability of the pixel to fall within a peak region or a background region. The two models were built from an input layer of 512 nodes, a hidden layer of 256 nodes, and an output layer of 1 node. Dropout of 0.5 was used in‐between layers, as well as a ReLu activation function. The sigmoid activation function was used for the output layer. The models were trained using binary cross entropy as a loss function over 100 epochs and batch size of 10. A binary response was finally obtained from the probability output by the model using a threshold value of 0.5.

The smartphone‐based application had been coded using JavaScript and React. The open‐source framework React Native had been used alongside Expo Go client app to develop an application available on both iOS and Android platforms.

## Conflict of Interest

Lucas Dosnon and Inge K. Herrmann declare inventorship on a patent application filed by ETH Zurich and Empa on the in‐pad menstruation blood analysis platform (L. Dosnon, I.K. Herrmann, A device for detection of biomarkers, EP23209241). The authors declare no other competing interests.

## Author Contributions

L.D., T.R., and I.K.H. performed conceptualization and methodology. L.D. and C.M. performed investigation. L.D. performed visualization. I.K.H. performed funding acquisition and supervision. L.D. and I.K.H. performed project administration, wrote the original draft, and wrote, reviewed, and edited the draft. T.R., C.M., and I.K.H. provided resources. L.D. performed validation, data curation, formal analysis, and software.

## Supporting information



Supporting Information

## Data Availability

The data that support the findings of this study are available from the corresponding author upon reasonable request.
