# Supplementary material for: A Wearable In‐Pad Diagnostic for the Detection of Disease Biomarkers in Menstruation Blood
Source: Adv Sci (Weinh). 2025 May 24;12(32):e05170. doi: 10.1002/advs.202505170 (PMC12407263; doi:10.1002/advs.202505170)
Supplement: Supplementary file 1 — Supporting Information [file ADVS-12-e05170-s001.pdf]

ADVANCED SCIENCE
Open Access

Supporting Information

for *Adv. Sci.*, DOI 10.1002/advs.202505170

A Wearable In-Pad Diagnostic for the Detection of Disease Biomarkers in Menstruation Blood

*Lucas Dosnon, Thomas Rduch, Charlotte Meyer and Inge K. Herrmann**

Supplementary information

A Wearable In-pad Diagnostic for the Detection of Disease Biomarkers in Menstruation Blood

*Lucas Dosnon[1,2,4,5], Thomas Rduch[2,3], Charlotte Meyer[1,2,4,5], Inge K. Herrmann[1,2,4,5]**

[1] Nanoparticle Systems Engineering Laboratory, Institute of Energy and Process Engineering (IEPE), Department of Mechanical and Process Engineering (D-MAVT), ETH Zurich, Sonneggstrasse 3, 8092 Zurich, Switzerland.

[2] Particles Biology Interactions Laboratory, Department of Materials Meet Life, Swiss Federal Laboratories for Materials Science and Technology (Empa), Lerchenfeldstrasse 5, 9014 St. Gallen, Switzerland.

[3] Department of Gynecology and Obstetrics (Frauenklinik), Cantonal Hospital St. Gallen (KSSG), Rorschacherstrasse 95, 9007 St. Gallen, Switzerland.

[4] The Ingenuity Lab, University Hospital Balgrist and University of Zurich, Forchstrasse 340, 8008 Zurich, Switzerland.

[5] Faculty of Medicine, University of Zurich, Rämistrasse 100, 8006 Zürich, Switzerland

*corresponding author: ingeh@ethz.ch, inge.herrmann@empa.ch, inge.herrmann@uzh.ch

+41 (0)58 765 7153

Establishment of LFA for CEA, CA-125 and CRP in serum and whole blood

LFA sensors were designed for the detection and semi-quantification of individual biomarkers in relevant biofluids and clinically relevant detection windows. Careful optimization of the LFA sensors was performed to achieve detection and semi-quantification in i) human serum, and ii) unprocessed human whole blood.

Direct measurement of CEA level in human serum

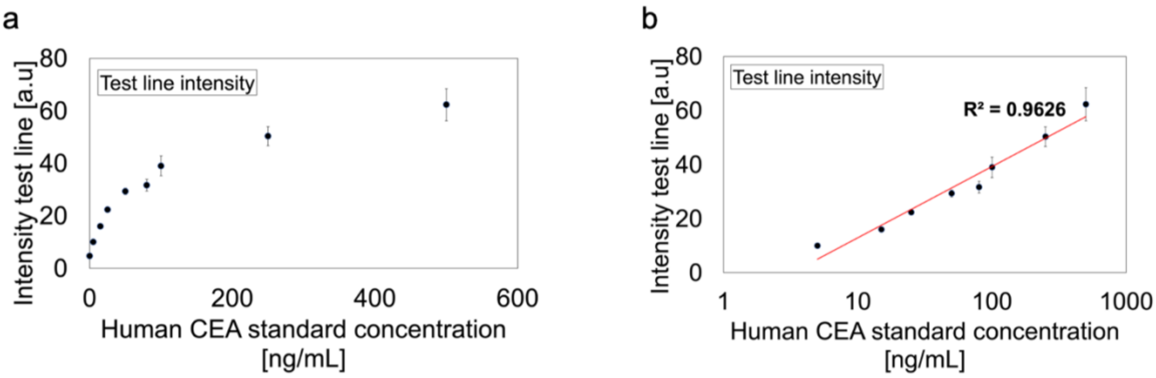

Figure S1: Detection and quantification of CEA using CEA LFA with human serum. a) A logarithmic response curve was observed with increasing concentration of CEA between 0 and 500 ng/mL. b) Considering a log scale, a linear response curve can be obtained on the entire range of concentration with a coefficient R^2 of 0.96.

Direct measurement of CRP level in human serum and human whole blood

The full CRP concentration range (0 to 500 µg/mL) is rarely measurable due to the hook effect. At concentrations above 10 µg/mL (Fig. S2 a,c), the test line intensity decreased while the CRP concentration increased, rendering interpretation of the test challenging due to false negatives. Using a sandwich assay-based line, a linear detection window was only obtained for concentrations of CRP in serum and blood between 0 and 10 µg/mL (Fig S2 a,b,c). This demonstrates the necessity to introduce a competitive assay-based line. In both human serum and human whole blood, the signal generated by this line allowed interpretation of the test results on the full CRP concentration range (0 to 500 µg/mL) without generating false negatives (Fig S2 d).

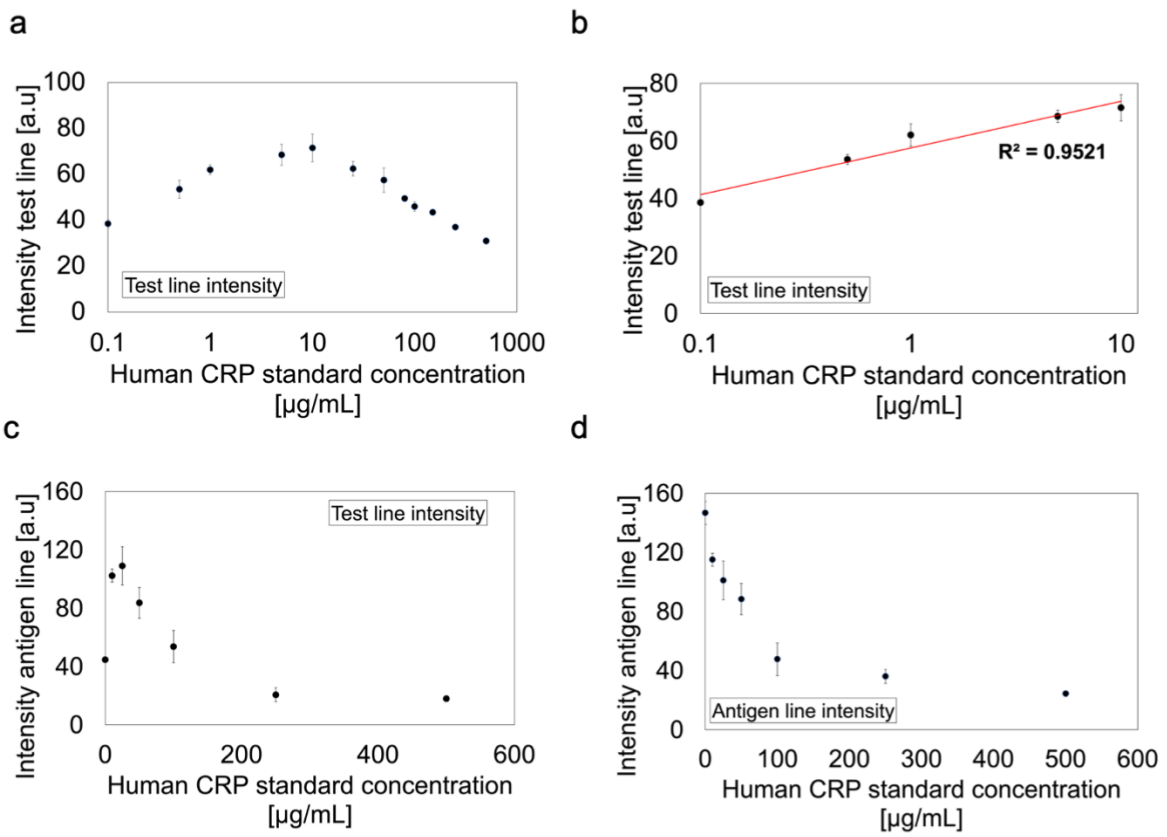

Figure S2: Detection and quantification of CRP using CRP LFA. a) Detection in human serum and b) human whole blood. a) The test line intensity response with increasing CRP concentration showed the occurrence of the hook effect at concentration of CRP in human serum above 10 µg/mL. b) A linear detection response was obtained between 0.1 and 10 µg/mL using the test line response with a coefficient R^2 of 0.95. c) The test line intensity response with increasing CRP concentration showed the occurrence of the hook effect at concentration of CRP in human whole blood above 10 µg/mL. d) The competitive assay-based line intensity response showed no occurrence of the hook effect with increasing concentration of CRP on the entire concentration range (0 to 500 µg/mL).

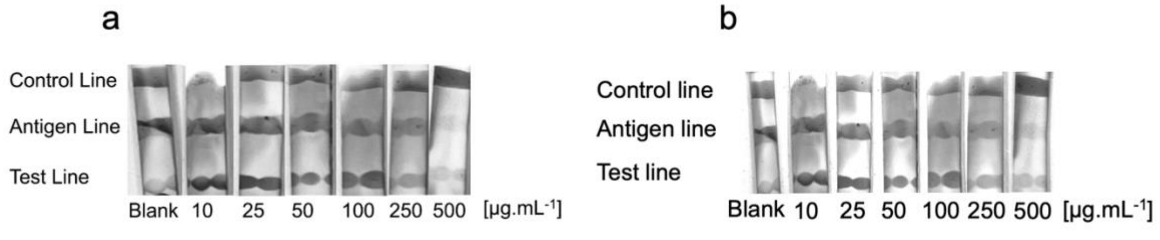

Figure S3: Comparison of CRP detection LFA readout color at two different time points. a) The image included in the original manuscript has been taken on the 17.04.2023. b) The image has been taken as comparison on the 01.07.2024.

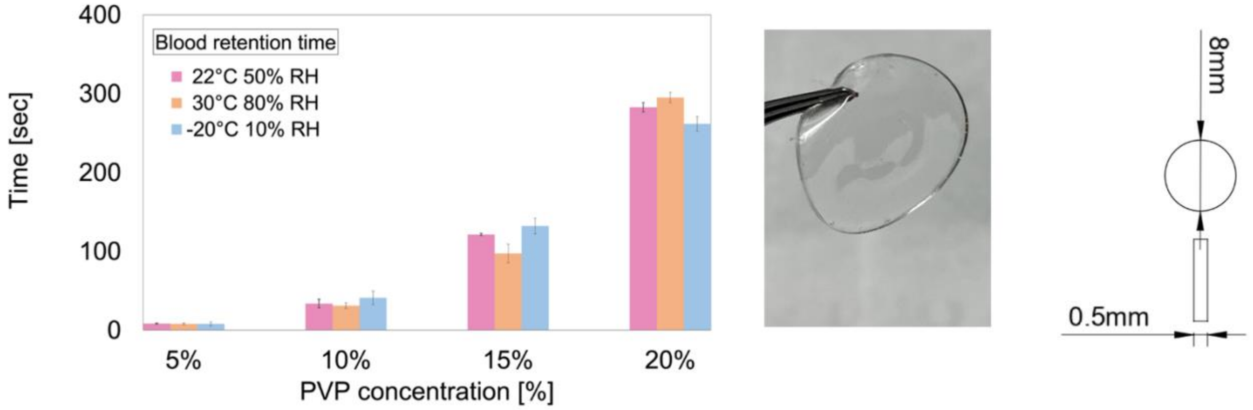

Figure S4: Dissolvable membrane concept and characteristics showing a controllable dissolving time based on PVP concentration.

	**Human body**	**Chair**
**Density**	1.060e+3 kg/m^3	560 kg/m^3
**Young's modulus**	2.890e+9 Pa	9.300e+9 Pa
**Poisson's ratio**	0.380	0.350
**Yield strength**	4.033e+7 Pa	4.660e+7 Pa

Figure S5: Mechanical properties for the materials used as part of the bodyweight distribution simulations shown on Fig 3i.

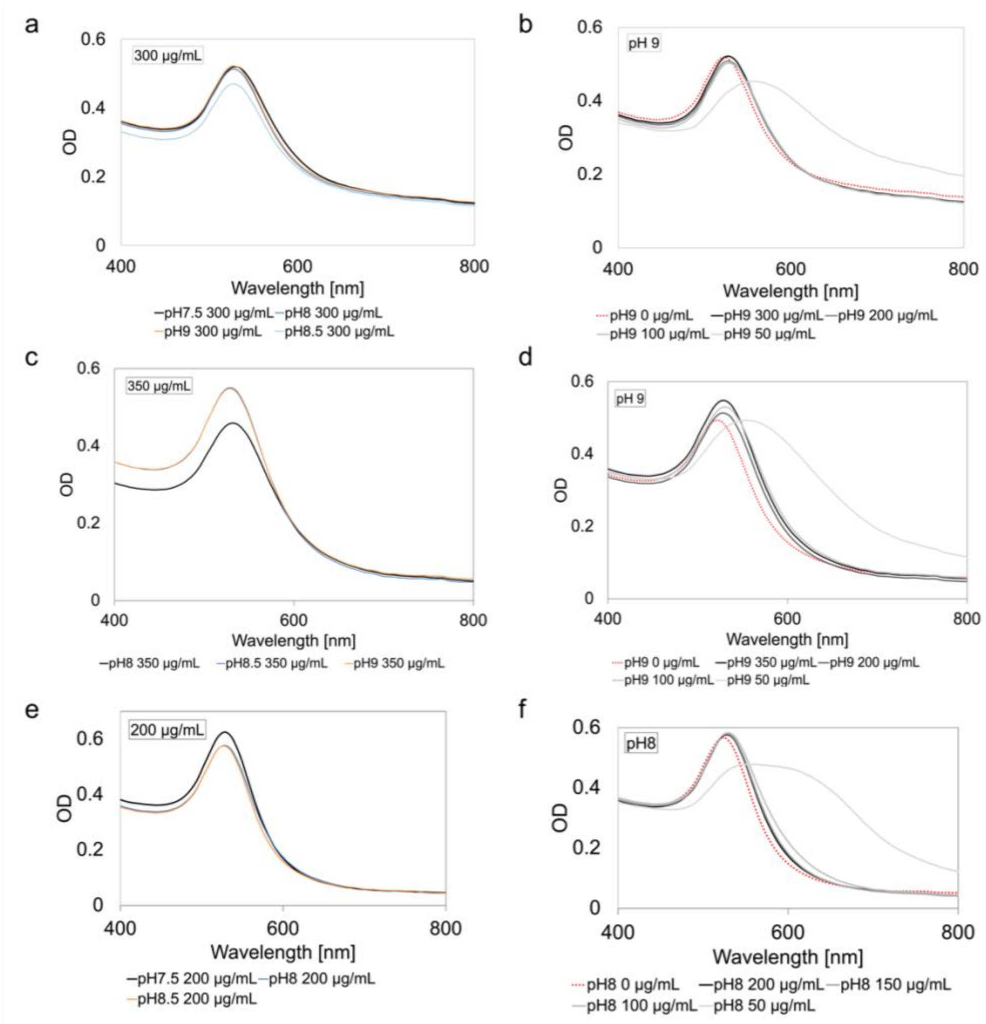

Figure S6: Optimization of nanoparticle solutions pH and antibody concentrations during nanoparticle-aggregation test for optimal conjugation and maximization of assay performances. a) Conjugation output at various pH of nanoparticle solution at the determined optimal concentration of CEA antibody solution. b) Conjugation output at various concentrations of CEA antibody solution for the determined optimal pH of nanoparticle solution. c) Conjugation output at various pH of nanoparticle solution at the determined optimal concentration of CA-125 antibody solution. d) Conjugation output at various concentrations of CA-125 antibody solution for the determined optimal pH of nanoparticle solution. e) Conjugation output at various pH of nanoparticle solution at the determined optimal concentration of CRP antibody solution. f) Conjugation output at various concentrations of CRP antibody solution for the determined optimal pH of nanoparticle solution.

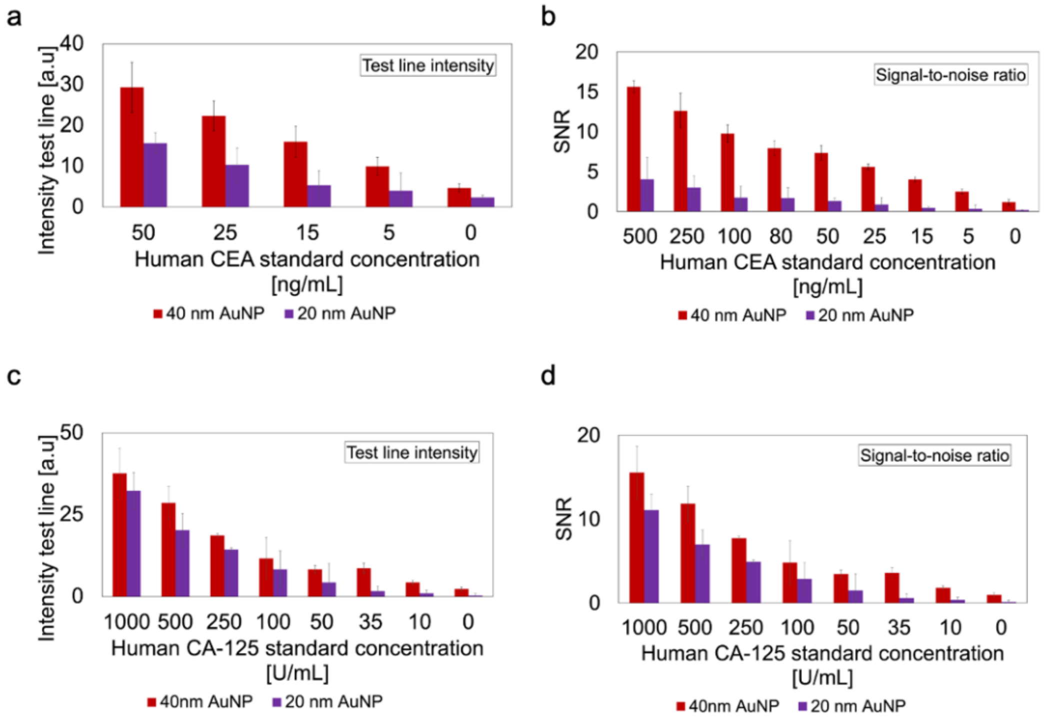

Figure S7: Comparison of signal intensities and signal-to-noise ratios (SNR) between 40 nm and 20 nm gold nanoparticles. a) Comparison of signal intensities for CEA detection. b) Comparison of SNR for CEA detection. c) Comparison of signal intensities for CA-125 detection. d) Comparison of SNR for CA-125 detection.
